# Bone mineral density measurement and osteoporosis treatment after a fragility fracture in older adults: regional variation and determinants of use in Quebec

**DOI:** 10.1186/1471-2474-6-33

**Published:** 2005-06-21

**Authors:** Alain Vanasse, Pierre Dagenais, Théophile Niyonsenga, Jean-Pierre Grégoire, Josiane Courteau, Abbas Hemiari

**Affiliations:** 1 Department of Family Medicine, Faculty of Medicine, Université de Sherbrooke, 3001 12th Avenue North, Sherbrooke (QC) Canada, J1H 5N4; 2 PRIMUS Group, Clinical Research Center, Sherbrooke University Hospital, Sherbrooke (QC), Canada; 3 Hôpital Maisonneuve-Rosemont, Université de Montréal, Montreal (QC), Canada; 4 Population Health Research Unit and Faculty of Pharmacy, Université Laval, Quebec (QC), Canada

## Abstract

**Background:**

Osteoporosis (OP) is a skeletal disorder characterized by reduced bone strength and predisposition to increased risk of fracture, with consequent increased risk of morbidity and mortality. It is therefore an important public health problem. International and Canadian associations have issued clinical guidelines for the diagnosis and treatment of OP. In this study, we identified potential predictors of bone mineral density (BMD) testing and OP treatment, which include place of residence.

**Methods:**

Our study was a retrospective population-based cohort study using data from the Quebec Health Insurance Board. The studied population consisted of all individuals 65 years and older for whom a physician claimed a consultation for a low velocity vertebral, hip, wrist, or humerus fracture in 1999 and 2000. Individuals were considered to have undergone BMD testing if there was a claim for such a procedure within two years following a fracture. They were considered to have received an OP treatment if there was at least one claim to Quebec's health insurance plan (RAMQ) for OP treatment within one year following a fracture. We performed descriptive analyses and logistic regressions by gender. Predictors included age, site of fracture, social status, comorbidity index, prior BMD testing, prior OP treatment, long-term glucocorticoid use, and physical distance to BMD device.

**Results:**

The cohort, 77% of which was female, consisted of 25,852 individuals with fragility fractures. BMD testing and OP treatment rates were low and gender dependent (BMD: men 4.6%; women 13.1%; OP treatment: men 9.9%; women 29.7%). There was an obvious regional variation, particularly in BMD testing, ranging from 0 to 16%. Logistic regressions demonstrate that individuals living in long term care facilities received less BMD testing. Patients who had suffered from vertebral fractures, or who had received prior OP treatment or BMD testing, regardless of gender, subsequently received more BMD testing and OP treatments. Furthermore, increasing the distance between a patient's residence and BMD facility precluded likelihood of BMD testing.

**Conclusion:**

BMD testing rate was extremely low but not completely explained by reduced physical access; gender, age, social status, prior BMD testing and OP treatment were all important predictors for future BMD testing and OP treatment.

## Background

Osteoporosis (OP) is defined as a skeletal disorder characterized by reduced bone strength which predisposes a person to an elevated risk of fracture [1]. This disease is an important public health problem in societies with an ever-increasing proportion of elderly people. It is projected that in Canada, approximately one in four women and one in eight men will suffer from OP during their lifetime [2]. Fragility fracture is the main adverse consequence of this disease.

Fragility fracture is defined by the World Health Organization as "*a fracture caused by injury that would be insufficient to fracture a normal bone...the result of reduced compressive and/or torsional strength of bone*" [3]. Clinically, a fragility fracture may be defined as a fracture "...*that occurs as a result of a minimal trauma, such as a fall from a standing height or less, or no identifiable trauma*" [4]. Typical fractures in patients with osteoporosis include vertebral (spine), proximal femur (hip), distal forearm (wrist) and proximal humerus [4,5].

The number of fragility fractures is expected to increase over the next 20 years in Canada; in 2041, the number of forecast hip fractures will be 88,124, a 3-fold increase from 1993–94 [6]. Hip and vertebral fractures are associated with a particularly high level of morbidity and mortality [7-10]. One-year survival rates following a hip fracture are reduced [11,12]. In addition, individuals who have already sustained one fracture are at high risk for recurrence [13-17].

Fragility fractures lead to extensive use of health care resources and high costs of care [18-23]. Since 1998 [24], the Osteoporosis Society of Canada recommends using bone mineral density (BMD) measurement to confirm diagnosis of osteoporosis [3]. Various drugs are considered effective for the treatment of osteoporosis; such as hormonal replacement therapy and bisphosphonates (*etidronate *and *alendronate*) [3,25]. During our study period, from January 1999 to December 2000, the province of Quebec's health insurance plan (RAMQ) was offering unrestricted coverage for osteoporosis treatments; namely hormone replacement therapy (HRT), the bisphosphonates *etidronate *and *alendronate*, the selective modulator of estrogen receptors raloxifene and *calcitonine *nasal spray [26].

In developed countries, awareness and use of clinical guidelines by physicians is low. This can be partially explained by the lack of uniformity in recommendations [27]. In a large retrospective study, Solomon et al [28] found that the following patient variables lowered the probability of a physician's adherence to guidelines and thus patients in these categories were less likely to receive either BMD testing or OP treatment: patients aged >74 years (odds ratio [OR] = 0.49; 95% confidence interval [CI]: 0.43 to 0.55); patients aged < 55 years (OR = 0.34; 95% CI: 0.28 – 0.42); men (OR = 0.17; 95% CI: 0.12 – 0.23); black patients (OR = 0.40; 95% CI: 0.34 – 0.47), and patients with multiple comorbidities (OR = 0.79; 95% CI: 0.69 – 0.89). In another study, patient variables such as female gender, glucocorticoid use, and receipt of care from a rheumatologist all increased the likelihood of receiving BMD [27].

The failure to adhere to OP diagnosis and treatment guidelines results in missed opportunities for preventing new fractures [19,21,22]. A review article examining OP investigation, treatment and interventions following a fragility fracture in different countries concluded that [29] "*Investigation of OP by bone mineral density was low: 14 to 16 studies reported investigation of less than 32% of patients*." This same study revealed that even when diagnosis of OP was confirmed, only a fraction of patients were receiving calcium and vitamin D supplements (8 – 62%) and bisphosphonates (0.5 – 38%). In Canada, the proportion of patients with fragility fractures who receive a BMD test or a physician diagnosis of OP ranges widely from 1.7 to 50% [21]. Moreover, only 5.2 to 37.5% of patients with fragility fractures receive pharmacotherapy (HRT, bisphosphonates or calcitonine) for their osteoporosis [21].

One of the factors contributing to low BMD testing in Canada may be the low geographical accessibility to specialized BMD equipment and trained personnel. This was well documented in two studies examining the patterns of use of BMD in Ontario [30,31]. In the first, the age-adjusted rate of BMD testing for the 1996–1998 time period in 49 Ontario counties varied from 0.2 to 47.1 per 1000 women [30]. Regional rate variation analyses of BMD performed during that same period indicated a 235-fold variation in BMD testing across counties, these rates being much higher in urban, south-central regions than in rural northern Ontario. According to this study, the large variation of access to BMD testing correlated directly with the location of BMD devices [32]. This urban/rural difference was also observed in a survey of Ontario general practitioners in which physicians with urban practices reported a higher use of BMD than their rural counterparts.

In 2001, 71 BMD devices were available in the province of Quebec; nearly half of these were located in Montreal. To our knowledge, regional variations in the diagnostic process and treatment of OP have not yet been studied within the province of Quebec.

The main objectives of this study were to estimate the BMD testing rate and OP treatment rate for people over 65 years of age who had already suffered from a fragility fracture, and to examine how the location of residency is related to BMD testing and OP treatment. We sought to identify potential predictors of BMD testing in the two years following a fragility fracture, as well as predictors of OP treatment in the year following the index fracture.

## Methods

### Study design

We conducted a retrospective population-based cohort study using data from the *Régie de l'Assurance Maladie du Québec *(RAMQ), the Quebec Health Insurance Board. Quebec is the second largest Canadian province with a population in 2001 of 7.4 million [33]. The RAMQ covers all Quebec citizens for physician services. For prescription drugs, the RAMQ covers people aged 65 years or more as well as welfare recipients and people not covered by private drug insurance.

### Studied population

The studied population consisted of all individuals 65 years or older for whom a physician claimed a consultation between 1 January 1999 and 31 December 2000 for one of the following fractures: vertebral (ICD-9 code 805), hip (ICD-9 code 820–821), wrist (ICD-9 code 814) or proximal humerus (ICD-9 code 812). The date of the first consultation for one of the above-mentioned fractures was defined as the index date. In order to include new cases only, we excluded all patients who had a previous vertebral, hip, wrist or humerus fracture during the 2-year period preceding the index date. We also excluded those who had a car accident or a work-related accident in the week preceding the index date since those fractures are less likely to be osteoporosis-related, and we excluded patients for whom data on place of residence was missing. The Northern Quebec region was excluded since it presents unique occupational and ethnic characteristics. Since October 1999, individuals living in a long-term care facility have been excluded from the RAMQ drug plan as prescription drugs are now provided free of charge by their facility [34]. These patients were therefore also removed from the analyses on OP treatment. Our algorithm was found to have a positive predictive value of 79% in an unpublished pilot project report [35].

### Data sources

Attributive and spatial data were used. Attributive data included all patient-data available in the RAMQ database. Data on accidents were obtained from the Quebec Car Insurance Board and from the Quebec Occupational Health and Security Board. Each patient was spatially referenced by his/her postal code of residence using data from DMTI Spatial [36] and from the Quebec Ministry of Health and Social Services [37]. The geographic coordinate system (GCS) used for cartographic presentation included at the end of the article was GCS North American 1983.

### Studied variables

Using encrypted health insurance numbers, we combined, at the patient level, the following RAMQ databases: the physicians' billing database, the prescription drugs database, the beneficiary database and the death register. The physicians' billing database contains data on the motive and date of consultation. The prescription drugs database contains the name and dosage of the drug, the duration of treatment, and the date prescriptions are filled. The beneficiary database provided data on age, sex, and date of death when applicable.

We assume that all BMD tests performed in the province of Quebec are billed to the RAMQ. If there was a physician claim for a dual-energy x-ray absorptiometry (DXA) procedure two years after the index date, the patient was considered to have undergone a BMD testing. During the study period, DXA was the only procedure covered by the RAMQ. We considered individuals to have received OP-related treatment if there was at least one pharmacy claim for a bisphosphonate (i.e. etidronate and alendronate), a HRT (for women), raloxifene, or calcitonine within one year after the index date. Other study variables included sex, age of the patient at the index date, site of fracture, type of beneficiary, comorbidity index, prior BMD testing, prior OP treatment, long-term glucocorticoid use, and distance from residence to the nearest BMD device.

Social status was divided into the following categories: residence in a public or subsidized private long-term care facility; residence at home and receiving a maximum guaranteed income supplement or social welfare; residence at home and receiving a partial guaranteed income supplement; and living at home and not receiving any guaranteed income supplement. We identified an individual's comorbidities by listing all medications other than OP drugs taken by the individual during the year prior to the index date [38]. Prior BMD testing was defined as a positive BMD test within the two years preceding the index date, while prior OP treatment was defined as the billing for at least one pharmacy claim for an OP treatment in the year preceding the index date. Long-term use of glucocorticoids was defined as glucocorticoid therapy (for at least 90 days within the 120 days before the index date) at a dose ≥ 5 mg per day [39]. Distance from residence to the nearest BMD device was defined as the aerial distance in kilometers (km) from the centroid (geometric center) of the individual's postal code to the centroid of the postal code of the nearest BMD device. For this variable, we used the cutpoints of 32, 64 and 105 km based on estimated transportation times of 60, 90 and 120 minutes respectively to cover these distances [40,41].

### Statistical analyses

We performed descriptive analyses by gender and place of residence and calculated age-adjusted BMD testing and OP treatment rates. When cell counts were large enough (>5), we used the Pearson χ^2 ^test for comparisons between proportions [42]; otherwise we used the Monte Carlo exact test. For comparison between means and medians, we used nonparametric tests (Kruskal-Wallis test, *k*-sample median test). We also calculated smoothed rates of BMD testing and OP treatments, and adjusted for gender, using the following geographically weighted regression (GWR) approach [43]. The 30 regional rates of BMD testing and the 30 regional rates of OP treatment (15 for each sex) were modeled as a function of gender, with parameters dependant on geographical coordinates of the region. For the cartographic representation and for gender comparison purposes, we grouped the 15 Quebec administrative regions in the same equally spaced intervals according to the BMD age-adjusted testing rates.

Logistic regression [44] analyses were performed by gender on BMD testing and OP treatment use. The potential patient-level predictors were age category (65–69, 70–74, 75–79, ≥ 80), site of fracture (vertebral, wrist, hip, humerus), type of beneficiary, comorbidity index, prior BMD, prior OP treatment, long-term glucocorticoid use, and finally, for the BMD testing model only, distance in kilometers from residence to the nearest BMD device. Statistical analyses were done using SAS [45], StatXact [46], and GWR [47]. Cartographic representations were done using ArcGIS [48].

### Ethical considerations

This project was approved by the *Comité d'Éthique de la Recherche sur l'humain *[Ethics Board] *de la Faculté de médecine de l'Université de Sherbrooke *and the *Commission d'accès à l'information du Québec*.

## Results

In 1999 and 2000, a total of 29,417 individuals aged 65 years or older consulted for a vertebral, hip, wrist or humerus fracture. Among them, 3140 had a fragility fracture within two years prior to the index date, 331 had an accident in the week preceding the index date and 94 had either incomplete data relating to the residence codes or were living in Northern Quebec. After exclusions, the cohort consisted of 25,852 individuals, 77% of which were women.

Characteristics of the population are presented along gender lines in Table 1. Almost all variables show statistically significant gender differences. The 2-year incidence rates of fragility fracture were 36 and 16 per 1000 inhabitants for women and men respectively. A very low rate of BMD testing at two years was found, particularly for men (4.6%), while a statistically significant higher proportion of men died during that same period as compared to women. Also, OP treatment one year after the fragility fracture was statistically lower for men (9.9%) than for women (29.7%). A higher proportion of women than men were recipients of the guaranteed income supplement and had prior BMD testing or prior OP treatment. Furthermore, both the mean and median distances from home to the nearest BMD device were lower for women than for men, and these differences were statistically significant (p < 0.0001). On the other hand, the long-term use of glucocorticoids failed to show any statistically significant gender differences.

**Table 1:** Characteristics of the population 65 years and older with a fragility fracture by gender

	Women	Men	p-value
*Population 65 years in 2000*	554,154	387,062	-
*Number of fragility fractures (per 1000)*	19,813 (36 ‰)	6039 (16 ‰)	< 0.0001
*Average age in years (standard deviation)*	78.7 (7.7)	76.6 (7.6)	< 0.0001
*Age category in years (%)*			< 0.0001
*65–69*	2842 (14.3)	1289 (21.3)	
*70–74*	3581 (18.1)	1326 (22.0)	
*75–79*	4312 (21.8)	1333 (22.1)	
≥*80*	9078 (45.8)	2091 (34.6)	
*Site of fragility fracture (%)*			< 0.0001
*Vertebral*	3006 (15.2)	1200 (19.9)	
*Wrist*	3391 (17.1)	734 (12.2)	
*Hip*	9194 (46.4)	2961 (49.0)	
*Humerus*	4222 (21.3)	1144 (18.9)	
Social status* (%)			< 0.0001
*Living in a LTCF*	1303 (6.6)	352 (5.8)	
*Living at home with maximum IS*	1840 (9.3)	234 (3.9)	
*Living at home with partial IS*	8757 (44.2)	2142 (35.5)	
*Living at home with no IS*	7913 (39.9)	3311 (54.8)	
*Comorbidity index – mean (quartiles)*	7.2 (3, 6, 10)	6.9 (2, 6, 10)	0.0065
*Prior BMD testing (%)*	1572 (7.9)	100 (1.7)	< 0.0001
*Prior OP treatment (%)*	3954 (20.0)	247 (4.1)	< 0.0001
*Long-term glucocorticoid use (%)*	407 (2.0)	132 (2.2)	0.5310
*Average distance to nearest BMD device*	20.3 km	23.4 km	< 0.0001
*Median distance to nearest BMD device*	3.0 km	3.5 km	< 0.0001
*BMD testing 2 years after fracture (%)*	2594 (13.1)	281 (4.6)	< 0.0001
*OP treatment one year after fracture (%)*	5889 (29.7)	596 (9.9)	< 0.0001
*Death 2 years after fracture (%)*	4507 (22.8)	2112 (35.0)	< 0.0001

* LTCF: Long-term care facility; IS: income supplement

**Figure 1:**
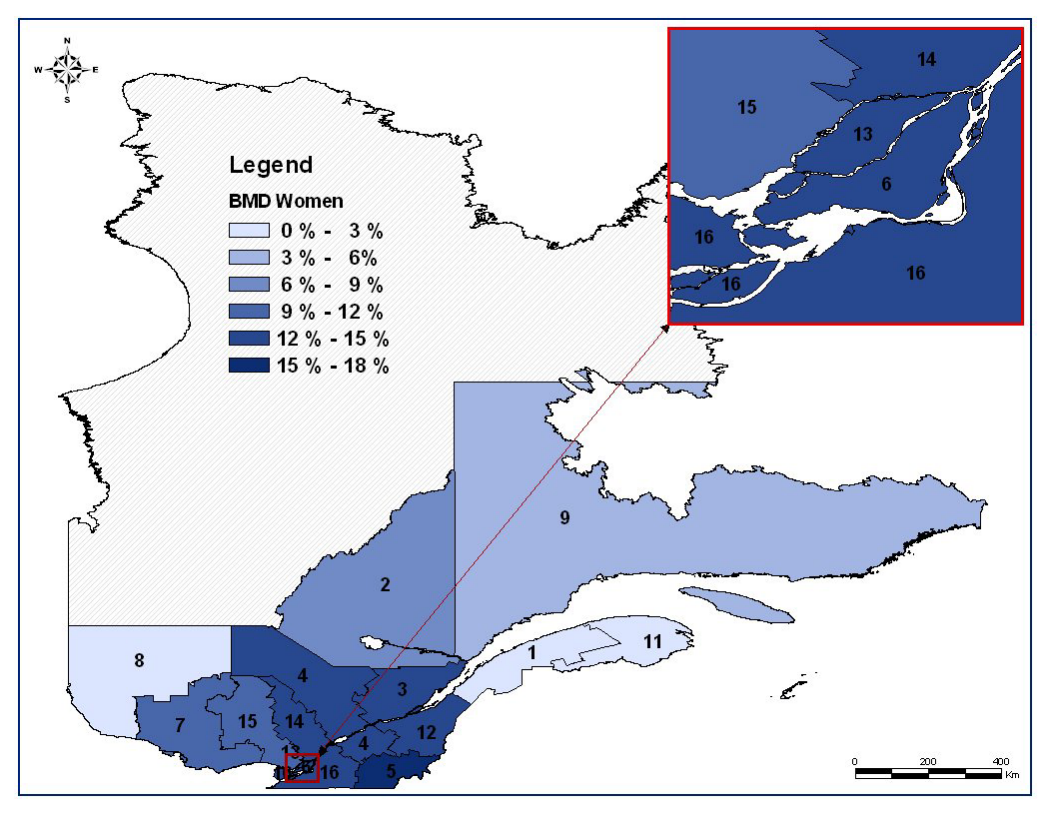
Map of the regional age-adjusted rates of bone mineral density (BMD) measurement two years after a fragility fracture in women after classification*. * Administrative regions: 1 : Lower St. Lawrence; 2 : Saguenay-Lac-St-Jean; 3: Quebec City; 4: Mauricie, Central Quebec; 5: Eastern Townships; 6: Montreal Center; 7: Outaouais; 8: Abitibi-Témiscamingue; 9: North Shore; 11: Gaspé, Magdalen Islands; 12: Chaudière-Appalaches; 13: Laval; 14: Lanaudière; 15: Laurentians; 16: Montérégie

**Figure 2:**
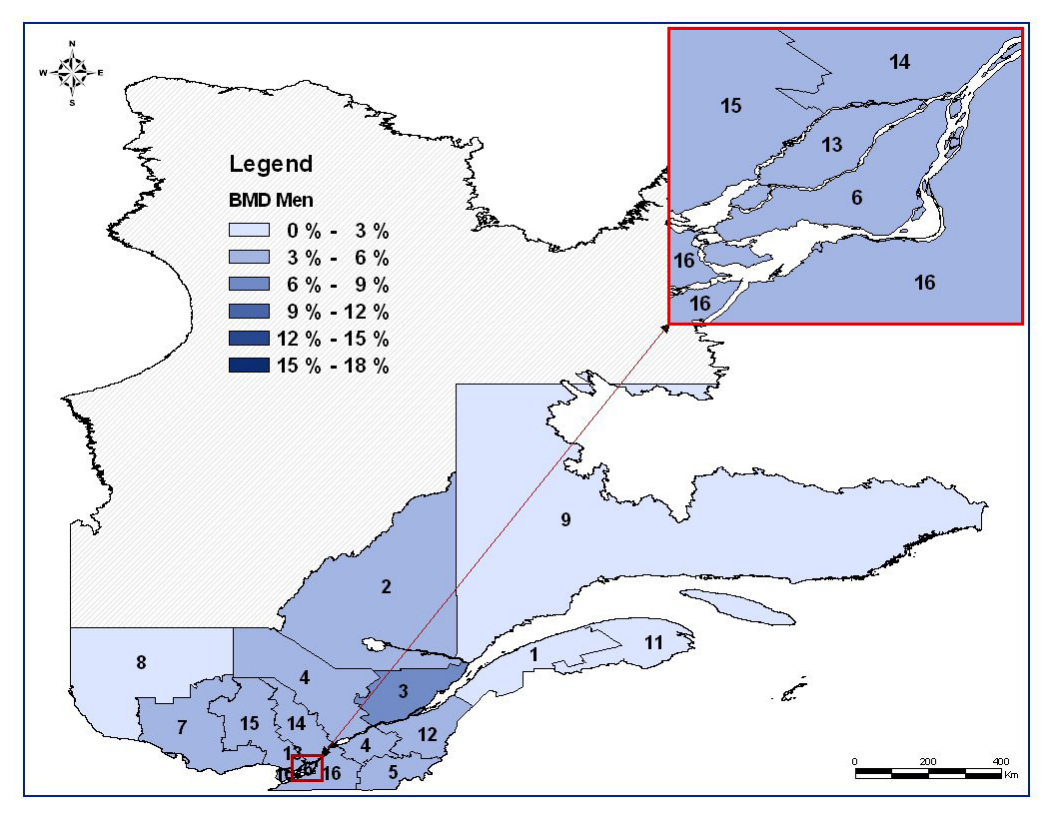
Map of the regional age-adjusted rates of bone mineral density (BMD) measurement two years after a fragility fracture in men after classification*. * Administrative regions: 1 : Lower St. Lawrence; 2 : Saguenay-Lac-St-Jean; 3: Quebec City; 4: Mauricie, Central Quebec; 5: Eastern Townships; 6: Montreal Center; 7: Outaouais; 8: Abitibi-Témiscamingue; 9: North Shore; 11: Gaspé, Magdalen Islands; 12: Chaudière-Appalaches; 13: Laval; 14: Lanaudière; 15: Laurentians; 16: Montérégie

Tables 2 and 3 display age-adjusted BMD testing rates and OP treatment rates after a fragility fracture, as well as mean and median aerial distance between residency and BMD device localization, for women and men, respectively. These tables also include the smoothed rates of BMD testing and OP treatment. These smoothed rates take into account the spatial nature of the data such as the neighboring regions. Figure 1 and Figure 2 display cartographic representations of age-adjusted BMD testing rates for women and men, respectively. There is a clear gender and regional variation in the use of BMD testing in favor of women and the most populated regions. For women, BMD testing rate varies from 0.3% in *Abitibi-Témiscamingue *region to 16.1% in *Quebec City *region, while for men it varies from 0% in *North Shore *region to 6.1% in *Quebec City *region. Regarding OP treatment, the regional variation is statistically significant but is less important than in the BMD testing rates. For women, OP treatment varies from 25.1% in *Outaouais *region to 39.9% in *Eastern Townships *region, whereas for men, OP treatment varies from 6.8% in *Laurentians *to 15.2% in *Eastern Townships *region.

**Table 2:** Women 65 years and older with fragility fractures in Quebec in 1999 and 2000: mean and median distances from residence to the nearest BMD device, age-adjusted bone mineral density measurement rate (BMD) with corresponding BMD smoothed rates (BMD SR), and age-adjusted osteoporosis treatment rate (OPT) with corresponding OP treatment smoothed rates (OPT SR) by administrative region

Region	Dist. BMD (km) mean (median)*	BMD (%)*	BMD SR (%)	OPT (%)*	OPT SR (%)
*Eastern Townships*	18.0 (12.9)	16.1	15.8	39.9	33.2
*Chaudière-Appalaches*	27.1 (19.7)	14.9	14.7	35.4	34.3
*Montérégie*	8.8 (3.8)	14.7	14.6	31.4	30.6
*Laval*	3.4 (3.3)	14.6	14.5	28.8	29.8
*Montreal-Center*	2.0 (1.7)	14.4	14.5	26.1	29.8
*Lanaudière*	22.6 (12.1)	13.4	13.0	29.2	29.3
*Quebec City*	10.0 (2.0)	13.2	13.7	29.3	31.8
*Mauricie, Central Qc*	19.8 (4.5)	12.9	12.9	29.5	29.6
*Laurentians*	29.9 (12.8)	11.7	12.0	30.8	28.7
*Outaouais*	24.5 (5.4)	9.4	9.8	25.1	28.0
*Saguenay-Lac-St-Jean*	26.3 (13.2)	8.8	9.7	38.9	34.1
*North Shore*	271.4 (314.4)	4.2	3.5	30.0	30.3
*Lower St. Lawrence*	85.7 (74.6)	2.3	3.7	34.4	33.6
*Gaspé, Magdalen Island*	331.0 (322.6)	1.1	1.9	32.3	31.9
*Abitibi-Témiscamingue*	88.9 (92.5)	0.3	3.9	25.9	27.4

* The difference between regions is statistically significant (p < 0.0001)

**Table 3:** Men 65 years and older with fragility fractures in Quebec in 1999 and 2000: mean and median distances from residence to the nearest bone mineral density device, age-adjusted bone mineral density measurement rate (BMD) with corresponding BMD smoothed rates (BMD SR), and age-adjusted osteoporosis treatment rate (OPT) with corresponding OP treatment smoothed rates (OPT SR) by administrative region

Region	Dist. BMD (km) mean (median)*	BMD (%)**	BMD SR (%)	OPT (%)*	OPT SR (%)
*Quebec City*	10.0 (2.0)	6.1	5.4	9.0	11.3
*Montérégie*	8.8 (3.8)	5.7	5.3	11.7	10.6
*Lanaudière*	22.6 (12.1)	5.5	5.0	13.3	10.0
*Eastern Townships*	18.0 (12.9)	5.4	5.3	15.2	12.1
*Laval*	3.4 (3.3)	5.4	5.3	9.9	10.2
*Montreal-Center*	2.0 (1.7)	4.9	5.3	6.9	10.2
*Mauricie, Central Qc*	19.8 (4.5)	4.5	4.8	10.5	10.4
*Chaudière-Appalaches*	27.1 (19.7)	4.4	5.1	13.6	12.5
*Outaouais*	24.5 (5.4)	3.9	3.7	8.5	9.5
*Laurentians*	29.9 (12.8)	3.6	4.2	6.8	9.5
*Saguenay-Lac-St-Jean*	26.3 (13.2)	3.5	3.9	12.5	11.7
*Gaspé, Magdalen Island*	331.0 (322.6)	1.7	1.1	9.1	11.7
*Abitibi-Témiscamingue*	88.9 (92.5)	0.7	1.9	10.9	9.7
*Lower St. Lawrence*	85.7 (74.6)	0.4	1.5	14.5	12.0
*North Shore*	271.4 (314.4)	0.0	0.8	12.2	11.7

* The difference between regions is statistically significant (p < 0.0001)

** The difference between regions is not statistically significant (p = .0578)

Logistic regression analyses (Table 4) show that women who had a prior BMD testing two years before the index date (OR: 2.45), who had a vertebral fracture (OR: 2.09), and who were exposed to OP treatment the year preceding the index date (OR: 1.51), were all more likely to be exposed to BMD testing. On the other hand, women aged 80 or older (OR: 0.17), women living in long-term care facilities (OR: 0.04), women with multiple comorbid conditions (OR: 0.98), and finally, women living over 105 km from the nearest BMD location (OR: 0.28), were less likely to undergo a BMD testing. Similarly, women who, prior to their fragility fracture, received an OP treatment (OR: 13.6) or a long-term glucocorticoid therapy (OR: 1.45), or who were tested for BMD (OR: 2.10), as well as those with a vertebral fracture (OR: 4.22), were more likely to obtain OP treatment in the year following the fracture. On the contrary, women aged 80 or older (OR: 0.60) were less likely to obtain such a treatment.

For men, logistic regression analyses (Table 5) show that those who had a prior BMD testing two years before the index date (OR: 5.15), those who received a long-term glucocorticoid treatment before the fracture (OR: 2.95), and those who had a vertebral fracture (OR: 2.77) were more likely to be exposed to BMD testing. On the other hand, men aged 80 or older (OR: 0.37), who lived in a long-term care facility (OR: 0.08), and finally, men living at more than 105 km from the nearest BMD location (OR: 0.14), were less likely to undergo a BMD testing. Likewise, men who received an OP (OR: 14.5) or a long-term glucocorticoid treatment (OR: 2.44) before their fragility fracture, and those who were exposed to BMD testing (OR: 3.80) prior to the fracture, were more likely to obtain an OP treatment in the following year. Also, those with multiple comorbid conditions (OR: 1.05), those with a vertebral fracture (OR: 5.15), older men, and those at home who were recipients of a partial guaranteed income supplement (OR: 1.27), were more likely to receive an OP treatment, whereas men with a wrist fracture (OR: 0.57) were less likely to receive an OP treatment.

**Table 4:** Predictors of receiving bone mineral density (BMD) measurement and predictors of osteoporosis (OP) treatment: Results from logistic regression models for women

	BMD testing		OP Treatment	
Covariates	Crude OR	Adjusted OR (95% CI)	Crude OR	Adjusted OR (95% CI)

*Age in years*				
*65–69*	1.00	-	1.00	-
*70–74*	0.71	0.79 (0.70; 0.89) ***	0.89	0.97 (0.85; 0.89 1.10)
*75–79*	0.42	0.52 (0.46; 0.59) ***	0.70	0.86 (0.76; 0.98) *
*≥80*	0.11	0.17 (0.15; 0.20) ***	0.40	0.60 (0.53; 0.67)***

*Fracture*				
Hip	1.00	-	1.00	-
Humerus	2.14	1.42 (1.26; 1.60)***	1.25	1.00 (0.90; 1.11)
Wrist	3.16	1.83 (1.62; 2.07) ***	1.52	1.18 (1.06; 1.32) *
Vertebral	2.17	2.09 (1.83; 2.38) ***	4.69	4.22 (3.80; 4.69) ***

*Social status*^§^				
*At home with no IS*	1.00	-	1.00	-
*At home with partial IS*	0.59	0.73 (0.67; 0.81)***	1.03	1.12 (1.03; 1.21) *
*At home with max IS*	0.31	0.46 (0.37; 0.56)***	0.76	0.93 (0.81; 1.07)
*Living in LTCF*	0.02	0.04 (0.02; 0.10)***	-	-

*Comorbidity index*	0.99	0.98 (0.97; 0.99) ***	1.07	1.02 (1.01; 1.03)***

*Prior BMD testing*	4.80	2.45 (2.16; 2.79)***	6.19	2.10 (1.82; 2.42)***

*Prior OP treatment*	2.45	1.51 (1.35; 1.68)***	16.8	13.6 (12.4; 15.0)***

*Long-term glucocorticoid use*	1.15	0.97 (0.71; 1.31)	3.93	1.45 (1.12; 1.89) *

*Distance from BMD device*				
≤ 32 km	1.00	-	-	-
*Between 32 and 64 km*	0.68	0.75 (0.62; 0.91) *		
*Between 64 and 105 km*	0.36	0.36 (0.25; 0.52)***		
*More than 105 km*	0.26	0.28 (0.19; 0.40)***		

* p < 0.05; ** p < 0.001; *** p < 0.0001

^§ ^LTCF: Long-term care facility; IS: income supplement

## Discussion

As in other studies, we found a low rate of BMD testing in high risk patients. Only 11% (13% of women and 5% of men) of patients having suffered from a fragility fracture were tested for osteoporosis or were tested to monitor response to therapy in the two years following their fracture. These results are comparable to the 0 to 32% [29] and 1 to 32% [27] frequencies of testing post-fragility fracture reported in large review papers. In our study, we found that BMD testing is not completely explained by a patient's physical access to densitometers. Gender, age and social status also seem to influence the rate of BMD testing.

Further, we found a moderate rate of OP treatment after a fragility fracture. A total of 25% (30% of women and 10% of men) of patients received an OP treatment in the year following the fracture. This is comparable with results from a review paper, which reported rates of bisphosphonate use ranging from 0.5% to 38% [29].

Finally, we found regional disparities in BMD testing according to the socio-demographic variables examined. For instance, age-adjusted BMD testing rates found in our study were statistically different among regions, varying from 0.3 to 16.1% for women and from 0 to 6.1% for men.

Hajcsar et al [49] had previously depicted gender differences in the investigation frequencies of osteoporosis. They reported a 1-year BMD testing rate of 24% for women and only 8.3% for men. These differences between our results and those reported by Hajcsar et al can be attributed to population and study design differences.

As reported in Jaglal [32], the accessibility to BMD testing seems to be strongly related to the use of a BMD device. Our results however show that the distance from residence to the nearest BMD device is neither the only nor the most important predictor of BMD testing. Younger age, previous BMD testing, and previous OP treatment use increased the likelihood of BMD testing, whereas social status, such as living in a long term care facility or being recipient of a guaranteed income supplement, decreased the likelihood of BMD testing. This last observation, not reported in other papers, was obtained by using a unique governmental data base that permits analysis of the above-mentioned social variables.

In the review papers cited above, patient characteristics were reported to influence BMD testing. In a study at two primary care practices affiliated with an academic medical center, Solomon et al found that some patient variables significantly lowered the probability of a physician's adherence to local guidelines (including BMD testing and OP treatment). Old age and young age as opposed to middle age, male sex, black race, and having more than one comorbid condition were associated with a decreased likelihood of undergoing a BMD testing or receiving an OP medication [28]. Our results are concordant with these reported findings concerning old age and gender but not regarding comorbidity. Comorbidity, as defined by the number of distinct medications taken in the year preceding the fracture, was negatively correlated with BMD testing for women, but was positively correlated with future OP treatment. In Solomon et al [28], these two outcomes were merged, although the authors argued that they had observed similar results when they were examined separately. With regards to comorbidity, the difference between our results and those reported by Solomon et al are likely due to differences in the methodology used and populations studied. For instance, comorbidity was defined differently in the two studies. Moreover, our analysis was population-based whereas Solomon et al studied patients seen in primary care practices affiliated with an academic medical center. Health care provided in academic centers may not be representative of the care provided to the general population.

Regarding therapy used to prevent recurrent fractures, our results showed that positive predictors for receiving treatment for both men and women were vertebral fracture, prior BMD testing, prior OP treatment, and long-term glucocorticoid use, as defined in the method section. Men with wrist fractures and women with hip or humerus fractures were less likely to be treated than individuals with fractures at other sites. These results are concordant with those of other studies [21,29]. The very low BMD testing rate in patients living in long-term care facilities suggests an important care gap in the management of OP in those facilities.

One might argue that patients may have died before receiving BMD testing or OP treatment. This would explain the lower rate of care observed in long term care facilities patients. In order to verify the stability of the associations between predictors and outcomes, we performed analyses on surviving patients during the two years following their fracture. These models showed similar results in predictors of BMD testing and OP treatment, except for site of fracture, for which we observed lower odds ratios. Exclusion of other predictors, such as prior BMD testing and prior OP treatment showed similar results between predictors and outcomes, except for long-term glucocorticoid use. Indeed, since patients who had received a long-term glucocorticoid therapy may have been already tested and/or treated for OP, the removal of these variables increased the associated OR.

We observed high death rates of 23% in women and 35% in men 2 years following fragility fracture. This is a 3-fold increase in death rates compared to those observed in the general elderly population for the year 2001 [50]. The 1-year death rate of the population aged 65 or older in Quebec in 2001 was 3.9% for women and 4.9% for men. An increase in mortality rates following fragility fracture was also reported in other studies [11,12].

A major strength of this study is its population based design. Moreover, to obtain a more comprehensive model explaining BMD use, we included a geographical distance variable in the modeling process. The major limitation of this study is inherent to the use of administrative databases. On the one hand, we may have underestimated the incidence of fragility fractures as some of these, such as vertebral fractures, are notoriously under-diagnosed by physicians [51]. We may also have overestimated incidence of fragility fractures since we inferred that all fractures not associated with a high velocity trauma were fragility fractures. However, in an unpublished pilot study, we found a positive predictive value of 79% for fragility fracture diagnosis based on this definition [35]. One of the limitations regarding the use of the guaranteed income supplement as a social status indicator includes a possible under-estimation of the number of beneficiaries in this category, as nearly 31% of individuals in Canada who are admissible for a guaranteed income supplement do not benefit from it [52]. Furthermore, as we have excluded fragility fractures within the 2 years prior to the index date we may have excluded at risk patients. Unfortunately, we are limited by the fact that data before the year 1997 are unavailable. It is well known that physician characteristics may affect the incidence of BMD testing [27,28]. Although we acknowledge the importance of studying physician characteristics in the modeling process, we were unable to retrieve such data for technical reasons.

## Conclusion

We found in our study that the use of BMD testing two years after a fragility fracture was extremely low despite strong clinical guideline recommendations. This finding was not completely explained by geographical distances depriving patients' physical access to densitometers. Previous medical conditions including comorbidities, use of glucocorticoid or OP therapy, as well as fracture site were other important predictors of BMD testing and OP treatment. We found that other socio-demographic factors, such as gender, age and social status, were also important predictors of care provision in patients having suffered from fragility fracture.

## Competing interests

This project has benefited from an unrestricted grant by Merck Frosst Canada Ltd. J.-P. Grégoire is a former employee of Merck Frosst Canada Ltd.

## Authors' contributions

AV, PD, TN and JPG conceived the study, and PD performed the literature review on osteoporosis. JC performed the analyses. AV, PD, TN, JPG and JC participated in the writing of the manuscript. AH made the maps.

**Table 5:** Predictors of receiving bone mineral density (BMD) measurement and predictors of osteoporosis (OP) treatment: Results from logistic regression models for men

	BMD testing		OP Treatment	
Covariates	Crude OR	Adjusted OR (95% CI)	Crude OR	Adjusted OR (95% CI)

*Age in years*				
*65–69*	1.00	-	1.00	-
*70–74*	0.97	0.96 (0.69; 1.33)	1.42	1.46 (1.06; 2.01) *
*75–79*	0.86	0.86 (0.61; 1.20)	1.75	1.64 (1.20; 2.25) *
*≥80*	0.34	0.37 (0.25; 0.54) ***	1.44	1.54 (1.14; 2.08)*

*Fracture*				
*Hip*	1.00	-	1.00	-
*Humerus*	1.40	1.09 (0.75; 1.58)	0.87	0.84 (0.60; 1.17)
*Wrist*	1.34	1.01 (0.65; 1.57)	0.50	0.57 (0.35; 0.91) *
*Vertebral*	3.53	2.77 (2.06; 3.72)***	5.49	5.15 (4.13; 6.42) ***

*Social status*^§^				
*At home with no IS*	1.00	-	1.00	-
*At home with partial IS*	0.79	0.81 (0.62; 1.06)	1.33	1.27 (1.04; 1.56) *
*At home with max IS*	0.79	0.77 (0.40; 1.51)	1.10	1.26 (0.77; 2.07)
*Living in LTCF*	0.05	0.08 (0.01; 0.57)*	-	-

*Comorbidity index*	1.03	1.00 (0.98; 1.02)	1.09	1.05 (1.04; 1.07)***

*Prior BMD testing*	9.71	5.15 (3.00; 8.85)***	15.8	3.80 (2.19; 6.58)***

*Prior OP treatment*	3.45	1.36 (0.83; 2.23)	26.1	14.5 (10.4; 20.1)***

*Long-term glucocorticoid use*	5.16	2.95 (1.75; 4.97)	7.82	2.44 (1.54; 3.86) **

*Distance from BMD device*				
≤ *32 km*	1.00	-	-	-
*Between 32 and 64 km*	1.03	0.99 (0.64; 1.52)		
*Between 64 and 105 km*	0.61	0.65 (0.30; 1.41)		
*More than 105 km*	0.14	0.14 (0.03; 0.55)*		

* p < 0.05; ** p < 0.001; *** p < 0.0001

^§ ^LTCF: Long-term care facility; IS: income supplement

## Pre-publication history

The pre-publication history for this paper can be accessed here:



## References

[B1] (2000). Osteoporosis prevention, diagnosis, and therapy. NIH Consens Statement.

[B2] Goeree ROB, Pettitt DB, Cuddy L, Ferraz M, Adachi J (1996). An assessment of the burden of illness due to osteoporosis in Canada. J Soc Obstet Gynaecol Can.

[B3] (1998). Guidelines for preclinical evaluation and clinical trials in osteoporosis. World Health Organization Geneva.

[B4] Brown JP, Josse RG (2002). 2002 clinical practice guidelines for the diagnosis and management of osteoporosis in Canada. CMAJ.

[B5] Rose SH, Melton LJ, Morrey BF, Ilstrup DM, Riggs BL (1982). Epidemiologic features of humeral fractures. Clin Orthop.

[B6] Papadimitropoulos EA, Coyte PC, Josse RG, Greenwood CE (1997). Current and projected rates of hip fracture in Canada. CMAJ.

[B7] Cauley JA, Thompson DE, Ensrud KC, Scott JC, Black D (2000). Risk of mortality following clinical fractures. Osteoporos Int.

[B8] Greendale GA, Barrett-Connor E, Ingles S, Haile R (1995). Late physical and functional effects of osteoporotic fracture in women: the Rancho Bernardo Study. J Am Geriatr Soc.

[B9] Cooper C (1997). The crippling consequences of fractures and their impact on quality of life. Am J Med.

[B10] Koval KJ, Zuckerman JD (1994). Functional recovery after fracture of the hip. J Bone Joint Surg Am.

[B11] Cummings SR, Melton LJ (2002). Epidemiology and outcomes of osteoporotic fractures. Lancet.

[B12] Cooper C, Atkinson EJ, Jacobsen SJ, O'Fallon WM, Melton LJ (1993). Population-based study of survival after osteoporotic fractures. Am J Epidemiol.

[B13] Klotzbuecher CM, Ross PD, Landsman PB, Abbott TA, Berger M (2000). Patients with prior fractures have an increased risk of future fractures: a summary of the literature and statistical synthesis. J Bone Miner Res.

[B14] Mallmin H, Ljunghall S, Persson I, Naessen T, Krusemo UB, Bergstrom R (1993). Fracture of the distal forearm as a forecaster of subsequent hip fracture: a population-based cohort study with 24 years of follow-up. Calcif Tissue Int.

[B15] Lauritzen JB, Schwarz P, McNair P, Lund B (1993). Radial and humeral fractures as predictors of subsequent hip, radial or humeral fractures in women, and their seasonal variation. Osteoporos Int.

[B16] Gunnes M, Mellstrom D, Johnell O (1998). How well can a previous fracture indicate a new fracture? A questionnaire study of 29,802 postmenopausal women. Acta Orthop Scand.

[B17] Cuddihy MT, Gabriel SE, Crowson CS, O'Fallon WM, Melton LJ (1999). Forearm fractures as predictors of subsequent osteoporotic fractures. Osteoporos Int.

[B18] Looker AC, Johnston CC, Wahner HW, Dunn WL, Calvo MS, Harris TB, Heyse SP, Lindsay RL (1995). Prevalence of low femoral bone density in older U.S. women from NHANES III. J Bone Miner Res.

[B19] Gardner MJ, Flik KR, Mooar P, Lane JM (2002). Improvement in the undertreatment of osteoporosis following hip fracture. J Bone Joint Surg Am.

[B20] Freedman KB , Kaplan FS, Bilker WB, Strom BL, Lowe RA (2000). Treatment of osteoporosis: are physicians missing an opportunity?. J Bone Joint Surg Am.

[B21] Papaioannou A, Giangregorio L, Kvern B, Boulos P, Ioannidis G, Adachi JD (2004). The Osteoporosis Care Gap in Canada. BMC Musculoskelet Disord.

[B22] Torgerson DJ, Dolan P (1998). Prescribing by general practitioners after an osteoporotic fracture. Ann Rheum Dis.

[B23] Simonelli C, Chen YT, Morancey J, Lewis AF, Abbott TA (2003). Evaluation and management of osteoporosis following hospitalization for low-impact fracture. J Gen Intern Med.

[B24] (1998). Consensus conference on menopause and osteoporosis. Canadian Society of Obstetrics and Gynaecology.

[B25] Kanis JA (1994). Assessment of fracture risk and its application to screening for postmenopausal osteoporosis: synopsis of a WHO report. WHO Study Group. Osteoporos Int.

[B26] Régie de l'Assurance-Maladie du Québec (2000). Liste des médicaments.

[B27] Morris CA, Cabral D, Cheng H, Katz JN, Finkelstein JS, Avorn J, Solomon DH (2004). Patterns of bone mineral density testing: current guidelines, testing rates, and interventions. J Gen Intern Med.

[B28] Solomon DH, Brookhart MA, Gandhi TK, Karson A, Gharib S, Orav J, Shaykevich S, Licari A, Cabral D, Bates DW (2004). Adherence with osteoporosis practice guidelines: a multilevel analysis of patient, physician, and practice setting characteristics. Am J Med.

[B29] Elliot-Gibson V, Bogoch ER, Jamal SA (2004). Practice patterns in the diagnosis and treatment of osteoporosis after a fragility fracture: a systematic review. Osteoporos Int.

[B30] Jaglal SB, McIsaac WJ, Hawker G, Jaakkimainen L, Cadarette SM, Chan BT (2000). Patterns of use of the bone mineral density test in Ontario, 1992–1998. CMAJ.

[B31] Ridout R, Hawker GA (2000). Use of bone densitometry by Ontario family physicians. Osteoporos Int.

[B32] Jaglal SB, McIsaac WJ, Hawker G, Jaakkimainen L, Cadarette SM, Chan BT (2000). Patterns of use of the bone mineral density test in Ontario, 1992–1998. CMAJ.

[B33] Institut de la statistique du Québec, population 2000 selon le groupe d'âge et le sexe. http://www.stat.gouv.qc.ca/donstat/societe/demographie.

[B34] (2004). Régie de l'assurance maladie du Québec. Statistiques annuelles 2003. Bibliothèque nationale du Québec,. http://www.ramq.gouv.qc.ca.

[B35] Bélanger A, Chiasson E, Godin S, Robidas F (2004). Recherche sur le potentiel de réduction des fractures de fragilisation chez les personnes de 50 ans et plus hospitalisés au CHUS. Dans le cadre du stage en santé communautaire 2004. Université de Sherbrooke Janvier.

[B36] (2000). DMTI Spatial Data Delivery System, CanMap Streetfiles and PostCode files. http://www.dmtispatial.com.

[B37] Ministère de la Santé et des Services sociaux. http://www.msss.gouv.qc.ca.

[B38] Schneeweiss S, Seeger JD, Maclure M, Wang PS, Avorn J, Glynn RJ (2001). Performance of comorbidity scores for confounding in epidemilogic studies using claims data. American Journal of Epidemiology.

[B39] McIlwain HH (2003). Glucocorticoid-induced osteoporosis : pathogenesis, diagnosis, and management. Preventive Medicine.

[B40] Scott PA, Temovsky CJ, Lawrence K, Gudaitis E, Lowell MJ (1998). Analysis of Canadian population with potential geographic access to intravenous thrombolysis for acute ischemic stroke. Stroke.

[B41] Winters RC, Hendey GW, Bivins H (1998). Helicopter versus ground ambulance transport is a helicopter actually faster?. Acad Emerg Med.

[B42] Conover WJ (1999). Practical nonparametric statistics.

[B43] Fotheringham AS, Brunsdon C, Charlton M (2002). Geographically Weighted Regression: the analysis of spatially varying relationships.

[B44] Hosmer DW, Lemeshow S (1989). Applied Logistic Regression.

[B45] SAS Systems for Windows. Release 8.02.

[B46] (2001). StatXact-5 for Windows. Statistical Software for Exact Nonparametric Inference. Cytel Software Corporation.

[B47] Charlton M, Fotheringham AS, Brunsdon C (2003). GWR 3.0.1. University of Newcastle, England.

[B48] ArcGIS. Release 82 ESRI, Redlands, CA.

[B49] Hajcsar EE, Hawker G, Bogoch ER (2000). Investigation and treatment of osteoporosis in patients with fragility fractures. CMAJ.

[B50] Institut de la statistique du Québec. Taux de mortalité selon le groupe d'âge et le sexe, Québec, 2001, 2002 et 2003. http://www.stat.gouv.qc.ca/.

[B51] Lenchik L, Rogers LF, Delmas PD, Genant HK (2004). Diagnosis of osteoporotic vertebral fractures: importance of recognition and description by radiologists. AJR Am J Roentgenol.

[B52] (2001). Chambre des communes du Canada. Le supplément de revenu garanti à la portée de tous : un devoir. Rapport du Comité permanent du développement des ressources huimaines et la condition des personnes handicapées.

